# A conceptual framework for developing a boundary-based confidentiality training programme for primary healthcare nurses managing patients living with HIV

**DOI:** 10.4102/curationis.v49i1.2793

**Published:** 2026-02-16

**Authors:** Ntombesitatu Qotoyi, Agrinette N. Madolo

**Affiliations:** 1 Department of Nursing, Faculty of Health Sciences, Walter Sisulu University, Mthatha, South Africa

**Keywords:** conceptual framework, confidentiality, training programme, professional nurses, HIV care

## Abstract

**Background:**

Loss to follow-up and treatment defaulting in HIV care led to hospitalisations for advanced complications, undermining retention strategies. Breaches of confidentiality compel patients into transferring clinics to conceal their HIV status. The shift from traditional nurse–patient relationships to team-based care, while improving comprehensive services, complicates confidentiality maintenance and can increase risks of unintended disclosures, eroding trust. Observations across ten community health centres with nurse-led HIV and AIDS services in the OR Tambo district revealed high rates of antiretroviral therapy (ART) defaulting. The absence of a formal confidentiality training framework leads to inconsistent practices and nonadherence to confidentiality guidelines.

**Objectives:**

This study aimed to develop a contextually relevant conceptual framework guiding the design and implementation of a boundary-based confidentiality training programme for HIV care providers.

**Method:**

Empirical data were collected by semistructured interviews with nurses across ten centres and analysed thematically Dickoff’s six factors, comprising agent, recipient, context, procedure, dynamics and terminus, was used to guide the conceptual framework and ensure relevance and feasibility.

**Results:**

Four themes and twelve sub-themes formed the basis for the framework’s six components. These components provide a systematic approach to addressing training elements aligned with programme goals.

**Conclusion:**

The developed framework offers a structured foundation for tailored confidentiality training, guided by Dickoff’s model. This approach aims to improve confidentiality adherence, foster patient trust and promote treatment retention among HIV clients.

**Contribution:**

The framework bridges research, practice and education by providing an evidence-based, practically applicable and educationally effective approach to confidentiality training, ultimately improving knowledge and understanding of HIV care.

## Introduction

Maintaining confidentiality is indeed crucial in healthcare, especially in the context of HIV management. Ensuring that patients’ sensitive information is protected helps prevent discrimination, stigma and social exclusion, fostering a trusting relationship between healthcare providers and patients, which is essential for encouraging honest communication and adherence to treatment plans leading to better health outcomes. This article focusses on describing the conceptual framework employed to identify training needs essential for developing a boundary-based confidentiality training programme tailored for professional nurses managing people living with HIV in primary healthcare (PHC) facilities. A significant challenge highlighted is the lack of comprehensive guiding frameworks for the effective implementation of confidentiality policies, guidelines and boundary-setting practices among healthcare teams. This gap contributes to issues such as clients defaulting on treatment and poor retention in care, which undermine the effectiveness of HIV management programmes. By establishing a structured conceptual framework, the article aims to provide a foundation for addressing these challenges, promoting ethical practice and enhancing confidentiality adherence within healthcare teams to improve patient outcomes.

Structuring the programme concepts serves as the foundation for training programme development. These concepts emerged from the analysis of collected data, ensuring they are based on real-world evidence. When selecting and defining these concepts, relationships between them are recognised and established based on prior research, relevant philosophical perspectives and professional experience. Describing the concepts in detail helps clarify their meanings and significance, thereby enhancing understanding and guiding effective implementation. As noted by Chinn and Kramer (2017), this process of elaborating concepts is essential to deepen their meaning and ensure they are meaningful within the context of the programme’s goals.

## Research methods and design

This article aimed to develop a boundary-based confidentiality training programme for professional nurses managing patients with HIV in PHC in the OR Tambo district. The conceptual framework was developed following qualitative data collection from 19 professional nurses using a semistructured interview guide, serving as a precursor to the training programme. To identify the concepts relevant for intervention, a systematic approach was adopted. This approach facilitated a comprehensive understanding of confidentiality issues, enabling targeted interventions to prevent boundary violations and strengthen confidentiality practices. The classification process involved systematically organising data and concepts related to confidentiality practices among professional nurses within HIV care multidisciplinary teams. This was achieved with the identification of key concepts pertinent to the research problem, allowing for a structured analysis of how confidentiality is maintained, perceived and potentially compromised in these settings. Using Dickoff et al.’s survey list provided a structured approach to identify the roles (agent), targeted participants (recipient), contextual setting, procedural steps, motivating factors (dynamics) and desired outcomes (terminus). This structured classification ensures clarity and coherence in translating research findings into practical training interventions.

### Ethical considerations

Ethical clearance to conduct this study was obtained from Walter Sisulu University, Faculty of Health Sciences Research Ethics & Biosafety Committee (No. 021/2022). The conceptual framework was developed by synthesising findings and concepts derived from empirical data. Ethical approval was obtained from the research ethics committee. Participants were informed about the study’s purpose and objectives before seeking their consent, in accordance with the principle of respect. Confidentiality and anonymity were preserved by not disclosing facility or participant names during data collection.

## Results

The results illustrate the development of the conceptual framework, including the identification and description of key concepts. Its operationalisation was guided by Dickoff, James and Wiedenbach (1968) practice-oriented theory. From themes and sub-themes, independent and dependent variables related to confidentiality practices were identified, forming the major concepts driving the content and methodology of the boundary-based confidentiality training programme.

### Process for identification of concepts for the development of a conceptual framework

Concepts are ideas or general principles used to understand a phenomenon and represent reality (Polit & Beck 2021. Ravitch and Riggan (2017) describe a conceptual framework as the process of recognising assumed connections between important variables or concepts to be examined. The reasoning behind these assumptions can be drawn from various sources, including previous research conducted by the researcher, provisional theories and established theoretical or empirical findings available in the existing literature. From a ‘mental map’ perspective (Van der Waldt 2020), a conceptual framework is an overarching structure that guides a research project. It serves as a mental roadmap, integrating the researcher’s personal experiences, research questions, selected methodologies and methods, data analysis procedures and anticipated outcomes. This comprehensive structure provides clarity and direction throughout the research process.

Jarabeen (2009) further refines this understanding by describing the conceptual framework as a network of interconnected concepts that illustrate their relationships within the phenomenon under investigation. This conceptual network guides the design of the boundary-based training programme by clarifying how these concepts interact and influence each other, ensuring that the training effectively addresses the complexities of maintaining confidentiality boundaries in healthcare practice. The process for conceptual analysis entailed the following steps:

- Identification of the research problem, which was conducted in phase 1 of the empirical qualitative study.
- Development of research questions and semistructured interview questions, designed to describe, explore and contextualise various aspects related to the research problem, such as confidentiality practices, challenges faced by nurses and perceptions of both healthcare providers and patients regarding confidentiality in HIV care.
- Determination of key concepts and variables, followed by defining the relationships between these concepts to understand their interactions within the context of confidentiality practices.
- Formulation of hypotheses on how the concepts are related, grounded in existing theory and prior research to provide a conceptual framework.
- Utilisation of the practice-oriented theory by Dickoff et al. (1968) as a foundational theory to best explain the phenomena observed, which helped in developing a comprehensive framework for understanding confidentiality practices in HIV care.

### Selection of main concepts for core competencies

The main concepts were selected from the themes and sub-themes as outlined in Table 1.

**TABLE 1:** Themes and sub-themes to outline main concepts for core competencies.

Themes	Sub-themes	Independent variables	Dependant variables	Exploratory relationship	Main concepts for core competencies	Conclusion statements
Theme 1: Description of confidentiality guidelines and policies in place for HIV management	1.1 Dynamics related to confidentiality on adherence to HIV medication	Level of confidentiality practices and privacy measures	Adherence to HIV medication	Effective confidentiality practices, and robust privacy measures are associated with improved adherence to HIV medication	Knowledge	Professional nurses with higher knowledge and a nuanced understanding are more likely to adhere to confidentiality.
	1.2 Insights into confidentiality in HIV management	Patient provider relationship dynamics	Degree of boundary flexibility or rigidity depending on relationships	Relationship closeness may challenge or reinforce boundary practices	Knowledge	
	1.3 Maintaining professional boundaries in HIV management	Organisational policies and support for confidentiality	Healthcare provider’s adherence to confidentialityprotocols	Strong policies promote consistent adherenceamong staff	Understanding	
Theme 2: Participants expressed mixed experiences about maintaining confidentiality for patients with HIV.	2.1 Resource limitations as barriers to ensuring confidentiality.	Work environment such as workload, space and staffing levels	Ability to maintain boundaries under stress andhigh workload	Work environment variations affect individuals’ capacity to maintain boundaries	Experience	Training, as a focussed intervention, is essential when there is a deficiency of knowledge and experience among employees. When integrated with existing experience, supported by a conducive work environment and structured within a clear organisational framework, targeted training can effectively bridge gaps in understanding confidentiality.
	2.2 Lack of knowledge in confidentiality	Level of staff training and education on confidentiality and professional boundaries	Quality and consistency of boundary setting behaviour	Higher level of training often leads to better boundary management	Knowledge	
	2.3 Establishing clear guidelines to facilitate confidentiality to promote adherence	Organisational structure andleadershipcommitment	Overall perception in HIV care services	Supportive culture promotes environments where confidentiality is prioritised	Understanding	
	2.4 The significance of consent as a facilitator to maintain confidentiality	The level of staff awareness and knowledge about confidentiality rights, legal and ethical guidelines	Healthcare provider adherence to confidentiality protocols and ethical guidelines	Higher awareness and knowledge lead to better adherence to confidentiality standards	Knowledge	
Theme 3: Participants raised concerns related to breaches of confidentiality and boundary violations	3.1 Breach of confidentiality related to nonprofessional staff’s inexperience	Experience inhealthcareconfidentialitypractices	Confidence and skill in establishing and maintaining boundaries	More experience can improve boundary setting	Knowledge	Knowledge and practical experience are essential for employees to minimise confidentiality breaches. Ongoing training and real-world application within a supportive environment strengthen security and protect sensitive information.
	3.2 Approaches to dealing with a breach of confidentiality	Organisational culture and leadership commitment to manage confidentiality breaches	Overall perception of confidentiality in HIV services	Supportive culture creates an environment where confidentiality is prioritised.	Understanding	
		Availability and use of secure systems to report breaches	Frequency of confidentiality breaches or incidents reported	Secure systems reduce breaches, improving privacy	Experience	
Theme 4: Suggestions to improve confidentiality of HIV care	4.1 Training and workshops are prerequisites to improve confidentiality	Training and education on confidentiality and professional boundaries	Healthcare provider’s ability to maintain confidentiality	Training enhances provider’s knowledge, leading to better boundary maintenance	Empowerment	Enhancing confidentiality in HIV care requires a multifaceted approach that includes targeted training and workshops to empower staff with essential knowledge and skills.
	4.2 Adequate infrastructure and resources for quality HIV care	Workload and staffing levels	Risk boundary crossing or blurring	High workload may lead to boundary issues because of time constraints	Knowledge	
	4.3 Focus on staff attitudes and behaviour to improve confidentiality	Personal attitudes and cultural beliefs of healthcare providers	Behavioural practices regarding boundaries	Personal beliefs influence how boundaries are maintained or crossed	Understanding	
		Quality of communication skills among healthcare providers and patients	Patient confidence in sharing sensitive information	Good communication fosters trust and openness, reinforcing confidentiality	Knowledge	

### Defining the main concepts for core competencies identified in themes and sub-themes

A concept definition involves offering a precise explanation that clarifies its meaning by connecting it to related ideas or observable characteristics, thereby facilitating understanding. Having a clear definition helps develop measurement instruments that accurately capture the concept and ensures that all stakeholders refer to the same phenomenon (Nevin & Smith 2019). The following concepts were defined:

#### Understanding

Understanding is the ability to comprehend information, integrate different pieces of knowledge and apply that understanding to accomplish tasks. Understanding extends beyond possessing a unique style of knowledge and is demonstrated by the ability to think flexibly and apply what has been learned across different contexts and areas of performance (González 2019). It encompasses both superficial and extensive levels of comprehension. Superficial understanding involves awareness of only the obvious or surface features of a concept without depth or thoroughness, whereas extensive understanding entails a deeper, more comprehensive grasp that incorporates complex knowledge aligned with development levels. According to Collins Dictionary (n.d.), deep understanding refers to complete and thorough knowledge or grasp of a subject. In this article, a boundary-based confidentiality training programme is developed to improve nurses’ understanding of the subject, aiming to foster extensive comprehension. By explicitly distinguishing between superficial, extensive and deep understanding, the framework provides a robust structure for designing training activities that cultivate not just rote memorisation but a comprehensive and nuanced comprehension of the material. To reinforce extensive understanding, the training goes beyond surface-level concepts, encouraging critical thinking and reflection on ethical principles and practical applications related to confidentiality.

#### Knowledge

Knowledge is the accumulation of facts, information or skills acquired through education or experience. It includes an understanding of facts, actions and ideas (Robbins et al. 2009). Several types of knowledge have been identified in scholarly literature. Polanyi (1966) described tacit knowledge as difficult to express or communicate, often gained through practice and personal experience. In contrast, explicit knowledge relates to understanding the relationships between causes and effects and is obtained through personal experience, social interaction and formal education (Starbuck 1992). Explicit knowledge can be easily shared because it involves negotiated meanings within social groups or communities of practice (Schalow 2013). Ambrosini and Bowman (2001) argued that tacit and explicit knowledge are interconnected and cannot be completely separated.

Across various disciplines, terms like skills, intuition, know-how, procedural knowledge, implicit knowledge and experiential knowledge describe tacit knowledge, highlighting its diverse conceptualisations. Anderson, Krathwohl and Bloom (2001) expanded on these ideas by defining four types of knowledge within a structured framework: factual knowledge, which includes the fundamental elements of a discipline necessary for problem-solving; conceptual knowledge, which involves understanding how different facts and elements relate and work together; procedural knowledge, which pertains to knowing how to perform specific tasks or methods and metacognitive knowledge, which encompasses awareness of one’s own thinking processes, learning strategies and understanding of the context or conditions under which cognitive tasks are performed.

This conceptual framework supports the development of a training programme designed to address both explicit and tacit knowledge acquisition. By understanding how these types of knowledge interact, the framework guides the creation of training that combines formal instruction with practical experience. This integrated approach instils a deeper, more durable understanding in participants, leading to more effective learning and application.

#### Confidentiality

Confidentiality in nursing is a fundamental component of ethical patient care, grounded in respecting patient autonomy and fostering trust. It involves safeguarding personal health information from unauthorised access or disclosure (Chiruvella & Guddati 2021). Confidentiality in HIV and AIDS care is vital as it maintains patients’ privacy and autonomy and is essential for establishing trust (Pera & Van Tonder 2018). All patient information is considered confidential, encompassing any details used to identify an individual. Confidentiality emphasises the importance of establishing clear boundaries concerning what information is to be kept confidential, who has access to it and under what circumstances. By setting these boundaries, confidentiality is maintained within defined parameters, which helps prevent breaches and fosters trust among stakeholders. Thus, confidentiality can be defined as the ethical and professional duty to safeguard sensitive information by setting and adhering to boundaries specifying the scope and conditions of its protection. In this article, this outcome served as the conceptual foundation for designing the training programme.

#### Experience

Experience refers to the fact or state of having gained knowledge through direct observation or participation in events or activities. The Cambridge Dictionary (2020) explains experience as the process of acquiring knowledge or skills by doing, seeing or feeling things or as something that happens and affects an individual. Healthcare professionals interact with people regarding their experiences, and their perceptions of these experiences influence their ability to recognise the needs of individuals seeking care (Paulsen 2020).

By enriching their perspectives, professionals can develop a deeper understanding of the principles at work when engaging with clients concerning their experiences. An expected outcome is that many professionals will be better equipped to counsel, guide and supervise clients whom they might otherwise struggle to help (Paulsen 2020). This outcome will be achieved because professionals can access more perspectives when reflecting on client interactions.

#### Empowerment

Empowerment serves as a key management strategy essential for fostering nurses’ professional development and has a beneficial impact on the standard of patient care (García-Sierra & Fernández-Castro 2018). Empowering professional nurse’s entails establishing a conducive work setting that provides access to essential information, resources and opportunities for ongoing professional growth. Both structural empowerment, which involves organisational backing, and psychological empowerment, reflecting internal motivation and confidence, promote positive experiences among nurses and ensure positive patient outcomes (Bahlman-van Ooijen et al. 2023). This conceptual framework for a confidentiality training programme for nurses managing patients with HIV emphasises the importance of empowering nurses to effectively maintain patient confidentiality. The programme’s success will be measured not only by participants’ knowledge acquisition but also by the demonstrable improvements in their confidence and ability to apply confidentiality principles in their daily practice.

### Application of the conceptual framework

The components of the practice-oriented theory, as outlined by Dickoff et al. (1968), conceptualises the framework supporting this study. These components provide a systematic approach to collecting data, describing and predicting nursing practice, thereby making the practice more purposeful by clearly defining the specific goal of developing the training programme. This framework analyses concepts and activities, ensuring alignment with the anticipated and desired outcomes. The systematic method makes nursing practice more intentional and focussed by clearly defining the specific objectives to be achieved (McEwan & Wills 2019). The six components are as follows:

- Agent (who performs the activities),
- Recipient (who is the recipient of the activities),
- Context (in what context are the activities performed),
- Dynamics (interaction, challenges, findings),
- Procedure (what is the guiding procedure or techniques of the activities), and
- Terminus (what are the outcomes, goals, or end results of the activities).

**FIGURE 1:**
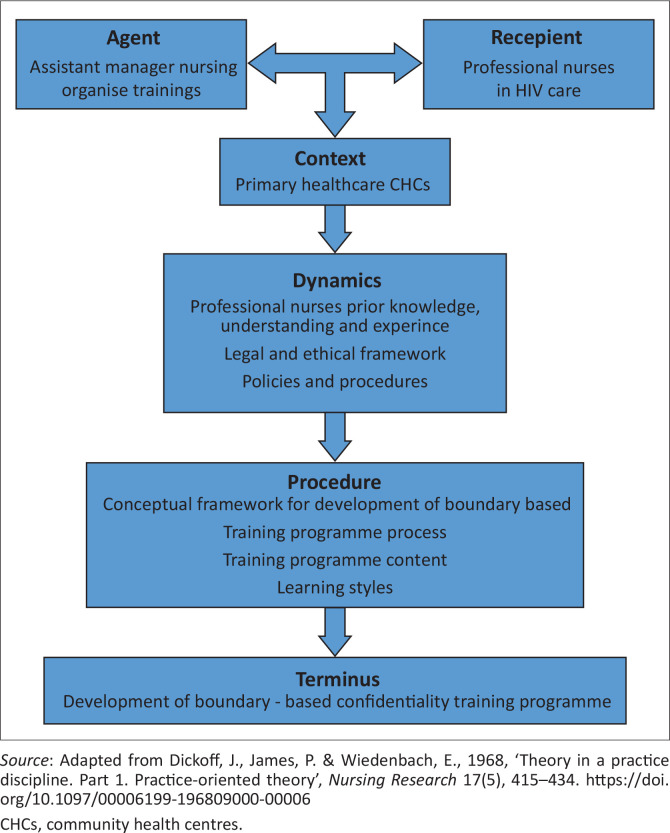
Reasoning map for the development of the conceptual framework.

According to Dickoff et al. (1968), the study should answer the six essential questions about the activities that must be performed, with the answers having cooperative implications for one another during the implementation of the training programme. The activities are described in the reasoning map in Figure 1.

### The agent: Who or what performs the activity?

Kamenye, Lipinge and Van Dyk (2016) describe an agent as someone who significantly influences outcomes, initiates actions and facilitates change, particularly in promoting new values, attitudes and behaviours. Dickoff et al. (1968) further define an agent as a motivating force behind achieving a goal, an active participant in producing a result. Preliminary study findings indicate nurses struggle to maintain confidentiality in HIV and AIDS care, demonstrating inconsistent handling of patient information. While policies and guidelines exist, there is lack of dedicated training programmes, resulting in a need for specific training content development.

Dickoff et al. (1968) describe the ‘agent’ as the individual responsible for achieving a nursing goal. This person, whether directly performing the action or overseeing its completion, has a clear intention to help the recipient reaching their goal. Crucially, the agent’s abilities, skills, training and experience are important factors to consider. In this study, an assistant nurse manager serves as the agent responsible for implementing the necessary training programme to address the identified confidentiality issues in HIV care. Is a professional nurse someone with 9 years of postregistration experience and a basic nursing qualification with the South African Nursing Council, including 3 years in a management position? They must hold a 1-year certificate in clinical nursing science and have 5 years of experience post-registration in clinical nursing science. They are hired as community health centre managers (ECDoH 2018).

According to Shandu (2008), the main areas of management for assistant nurse managers are as follows:

- Ensuring quality patient care while managing resources effectively and fairly, which are operational, human resources and finance of the nursing department.
- Ensuring compliance with professional and ethical practice.
- Training staff on updates and providing appropriate coaching.
- Conducting in-depth programme reviews.
- Problem-solving.

The assistant nurse manager plays a crucial role in identifying and addressing skills gaps within the nursing workforce. They conduct annual skills audits, analyse the results to determine training needs and formally submit these needs to human resource development. These documented training needs are then incorporated into the workplace skills plan (WSP), ensuring training schedules align with the organisation’s annual financial year. The WSP submission provides valuable input to the relevant Sector Education and Training Authority (SETA) regarding the industry’s skills needs and future development requirements. In the context of this study, the assistant nurse manager is responsible for organisational trainings.

### The recipient: Who or what is the recipient of the activity?

In this study, the recipients of the training programme are professional nurses providing HIV and AIDS services in PHC facilities who receive the boundary-based confidentiality training. Following Dickoff et al. (1968), these nurses receive the training activities designed to enhance their knowledge, skills, abilities and attitudes regarding confidentiality in HIV and AIDS care. The programme aims to address identified gaps in existing practices, building upon their existing knowledge from the basic training in ethics and professional practice experience. The recipients are expected to have a deep understanding of confidentiality and boundary setting to effectively transfer this knowledge to less experienced professional nurses and non-professional staff.

#### The context: In what context is the activity performed?

The environment in which an activity takes place is crucial (Dickoff et al. 1968). Nghipondoka-Lukolo and Charles (2015) define context as the situation, framework or environment surrounding an activity. This training programme focusses on nurses working in community healthcentres within the PHC system of the OR Tambo District Municipality, specifically those facilities providing HIV and AIDS services. The context of the activity, in this case, public PHC facilities, operates within a legal and ethical framework, policies and procedures and in an environment that need to be conducive with resources and support to maintain confidentiality.

The legal framework includes the Constitution of the Republic of South Africa, the *Health Act*, the *Nursing Act*, the South African Nursing Council rules and regulations and policies and procedures on confidentiality in HIV management. The findings revealed that nurses work within their profession’s legal and ethical framework, where the scope of practice is considered during delegation and task-shifting of duties. Ethical issues, including privacy, confidentiality, justice, beneficence and informed consent, should be adhered to by institutions in all their activities. Hamid et al. (2016) concluded that management should ensure a suitable and safe working environment for nurses by providing opportunities for professional development to improve their competency.

#### Policies and procedures

Professional nurses’ confidentiality is guided by policies and procedures in the institution where the training programme is to be implemented. The previously described qualities and characteristics of a professional nurse enable the nurse to effectively maintain confidentiality while caring patients with HIV and AIDS. Policies and procedures formulated in the institutions, in line with the state’s legal framework, are utilised by nurses to achieve set objectives. These policies and procedures should benefit the agent and the patient, ensuring quality care. When health professionals understand the standards and processes in the institution, they are more likely to facilitate the effective implementation of policies and guidelines. Training will promote communication of these policies and guidelines on confidentiality.

#### Environment, resources and management support

Implementing an effective training programme requires a conducive working environment to achieve optimal results. No healthcare facility can function effectively without adequate space, human resources and financial resources. Therefore, effective resource management is vital for healthcare institutions in achieving quality HIV services. The findings of the preliminary study revealed inadequate staff and material resources, including mobile screens for creating privacy, which affects confidentiality in HIV care in PHC facilities. Furthermore, professional nurses indicated a lack of management support and limited awareness of policies and guidelines on confidentiality or the content of in-service training. Despite these challenges, professional nurses must create a conducive environment for privacy and confidentiality to improve the quality of HIV care.

#### The dynamics: What is the energy source for the activity?

According to Dickoff et al. (1968), dynamics are the energy sources of the activities within an individual or the internal motivational factors promoting success. In this article, the dynamics include the barriers to maintaining confidentiality as experienced by nurses during HIV care. These refer to the guiding forces that motivate the direction towards change and development. Based on the findings of the main study, facilitators of confidentiality exist, but nurses’ attitudes and behaviour affect their practices. Nurses expressed concerns about the attitudes of their peers towards training, noting that poorly managed attitudes may hinder the implementation of the training programme and discourage participation. Strategies to address these factors must be developed to ensure the successful implementation of the training programme.

#### The procedure: What is the guiding procedure of the activity?

Procedures report challenges or guide actions for implementing a policy or programme (Nangombe & Justus 2016). A procedure is further described as an orderly way of completing tasks, which is also regarded as a rule guiding the activity, comprising devices and protocols enabling the agent to achieve the goal (Dickoff et al. 1968). In this article, procedure is the training programme that will be developed and implemented at PHC facilities in the OR Tambo District Municipality. The primary purpose is to provide professional nurses with the knowledge, skills and abilities to maintain confidentiality in HIV care. The procedure outlines conducting needs assessment, developing a training content based on the independent variables and dependent variables identified in the themes and sub-themes, the development, implementation and evaluation techniques, including programme content, learning objectives and learning outcomes. The programme’s content will be derived from the confidentiality attributes. The learning content aimed to improve professional nurses’ knowledge and understanding of maintaining confidentiality in HIV care (Mothiba & Jooste 2013).

### The terminus: What is the endpoint of the activity?

The sixth aspect on the survey list of the theory in a practice discipline by Dickoff et al. (1968) is the terminus, or the endpoint, of the activity. The article aimed to develop a conceptual framework, guiding the development of a boundary-based confidentiality training programme. This goal has been achieved, as the conceptual framework has been successfully developed, including guiding steps to facilitate the creation of the training programme. The outcome of this will be the development and implementation of a boundary-based confidentiality training programme.

## Conclusion

The article describes the concept classification process used to develop the conceptual framework, highlighting how concepts were identified and defined based on the independent and dependent variables derived from empirical study results. The six elements of the survey list facilitated the organisation of these concepts into coherent and understandable categories, which were essential for developing the training programme. Additionally, the six factors of Dickoff et al.’s (1968) practice-oriented theory agent, recipient, context, procedure, dynamics and terminus were employed to elucidate the roles and interactions within the training framework.
